# Automatic Sleep-Stage Scoring in Healthy and Sleep Disorder Patients Using Optimal Wavelet Filter Bank Technique with EEG Signals

**DOI:** 10.3390/ijerph18063087

**Published:** 2021-03-17

**Authors:** Manish Sharma, Jainendra Tiwari, U. Rajendra Acharya

**Affiliations:** 1 Department of Electrical and Computer Science Engineering, Institute of Infrastructure, Technology, Research and Management (IITRAM), Ahmedabad 380026, India; jainendra.tiwari.17e@iitram.ac.in; 2 School of Engineering, Ngee Ann Polytechnic, Singapore 599489, Singapore; aru@np.edu.sg; 3 Department of Bioinformatics and Medical Engineering, Asia University, Taichung 41354, Taiwan; 4 School of Management and Enterprise, University of Southern Queensland, Springfield 4300, Australia

**Keywords:** sleep stage, classification, electroencephalogram (EEG), polysomnogram (PSG), wavelet filters, sleep disorders

## Abstract

Sleep stage classification plays a pivotal role in effective diagnosis and treatment of sleep related disorders. Traditionally, sleep scoring is done manually by trained sleep scorers. The analysis of electroencephalogram (EEG) signals recorded during sleep by clinicians is tedious, time-consuming and prone to human errors. Therefore, it is clinically important to score sleep stages using machine learning techniques to get accurate diagnosis. Several studies have been proposed for automated detection of sleep stages. However, these studies have employed only healthy normal subjects (good sleepers). The proposed study focuses on the automated sleep-stage scoring of subjects suffering from seven different kind of sleep disorders such as insomnia, bruxism, narcolepsy, nocturnal frontal lobe epilepsy (NFLE), periodic leg movement (PLM), rapid eye movement (REM) behavioural disorder and sleep-disordered breathing as well as normal subjects. The open source physionet’s cyclic alternating pattern (CAP) sleep database is used for this study. The EEG epochs are decomposed into sub-bands using a new class of optimized wavelet filters. Two EEG channels, namely F4-C4 and C4-A1, combined are used for this work as they can provide more insights into the changes in EEG signals during sleep. The norm features are computed from six sub-bands coefficients of optimal wavelet filter bank and fed to various supervised machine learning classifiers. We have obtained the highest classification performance using an ensemble of bagged tree (EBT) classifier with 10-fold cross validation. The CAP database comprising of 80 subjects is divided into ten different subsets and then ten different sleep-stage scoring tasks are performed. Since, the CAP database is unbalanced with different duration of sleep stages, the balanced dataset also has been created using over-sampling and under-sampling techniques. The highest average accuracy of 85.3% and Cohen’s Kappa coefficient of 0.786 and accuracy of 92.8% and Cohen’s Kappa coefficient of 0.915 are obtained for unbalanced and balanced databases, respectively. The proposed method can reliably classify the sleep stages using single or dual channel EEG epochs of 30 s duration instead of using multimodal polysomnography (PSG) which are generally used for sleep-stage scoring. Our developed automated system is ready to be tested with more sleep EEG data and can be employed in various sleep laboratories to evaluate the quality of sleep in various sleep disorder patients and normal subjects.

## 1. Introduction

Sleep is indispensable for maintaining optimal health and well-being. Getting adequate sleep is equally crucial like daily exercise and balanced diet. According to health professionals, few of many benefits of getting good sleep include elevated productivity, improved calorie regulation, better concentration, reduced heart disease risks, superior athletic performance, increased social and emotional intellect and averting depression [1]. Considering an average human taking a sleep of 7–8 h per day, almost one-third of our life is spent sleeping. Our health is largely impacted by the quality of sleep we get. Any sleep related disorder directly affects our mental, physical and social as well as emotional well-being. According to International Classification of Sleep Disorders, Second Revision (ICSD-2) [2], sleep disorders are widely categorized into major categories like insomnia, parasomnia, hypersomnia of central origin, sleep-related disorder, circadian rhythm sleep disorder, sleep-related movement disorders and other disorders which also includes those caused due to some medical or psychological conditions [3]. Among them, insomnia is found to be the most prevailing sleep disorder [4]. Insomnia is described as a condition where one finds it extremely difficult to fall asleep and/or stay asleep. Symptoms of insomnia include fatigue, daytime sleepiness, cognitive impairment, irritability, impulsiveness or aggression. The person lasting in this condition for more than 4 weeks is diagnosed as suffering from insomnia. Based on several studies conducted in countries like the USA (3161 patients), Canada (5622 patients) and the UK (2363 patients), the reported prevalence of insomnia is around 35–37% [5]. In general, around 25–30% of the total population experience symptoms of insomnia. If patients with co-morbid conditions are also considered, this number may reach above 50% based on the type and severity of disease [5]. Bruxism is a movement disorder that is characterized by involuntary grinding, gnashing or clenching of teeth. Bruxism is listed in the ICSD and is the third most common form of sleep disorder after sleep talking and snoring [6]. There are mainly two classes of bruxism namely awake bruxism and sleep bruxism which refer to bruxism during awake stage and sleep stage, respectively. Pervasiveness of awake bruxism and sleep bruxism in the adult population is about 20% and 80%, respectively [7]. Studies have shown that awake bruxism is more dominantly observed in females as compared to males, whereas there is no such bifurcation in case of sleep bruxism [8]. Narcolepsy is a long-term rapid eye movement (REM) sleep disorder listed in ICSD and is characterized by irresistible deep sleep attacks during daytime which often occurs with or without cataplexy and hypnagogic hallucinations [9]. The cataplexy refers to the sudden loss of muscle power. The cyclic alternating pattern (CAP) sleep database also contains forty patients suffering from nocturnal frontal lobe epilepsy (NFLE) which accounts for 37% of the complete database. The NFLE is a sleep disorder of heterogeneous etiology [10] and is mainly characterized by epileptic seizures occurring due to the frontal lobe mainly during night (nocturnal) sleep. There are no such epidemiological data available for prevalance of NFLE as many cases of NFLE are misdiagnosed as parasomnias, especially when children are considered [11]. The database contains 10 patients suffering from periodic leg movement (PLM). The PLM refers to sleep disorder involving repetitive and rhythmic flexing or jerking of legs for about 20–40 s over a certain interval of sleep duration. This disorder has more prevalence than epilepsy [12]. This database contains 22 patients suffering from REM behaviour disorder (RBD). It is a parasomnia which is characterized by lack of normal muscular tension and other abnormal behaviour like enactment of dream during REM sleep. A study conducted by Ohayon et al. [13] showed the estimated prevalence of RBD to be 0.5%. Majority of RBD patients happen to be elderly males with age between 40 and 70 years [14]. Traditional diagnostic procedure for the above mentioned sleep disorders include night-long polysomnographic (PSG) analysis. It is a time-consuming, labour-intensive process and prone to human errors as long hours of continuous evaluation are required. Thus, an automated classification of sleep stages will help to overcome these drawbacks. Sleep scoring is an effective indicator and can help in the detection of various sleep related disorders.

The sleep-stage scoring is mostly done as per the rules presented by Rechtschaffen and Kales in 1968 [15]. According to R & K rules, sleep is mainly categorized into two stages namely rapid eye movement (REM) sleep and non-rapid eye movement (NREM) sleep. NREM sleep stage accounts for 75–80% and REM sleep stage usually lasts for around 20–25% of total sleep duration. NREM stage is further categorized into four sleep stages namely stage-1, 2, 3 and 4. Thus, a total of five sleep stages are known (1, 2, 3, 4 and REM).

Several studies have been proposed for the automated sleep stages classification. Recently, Loh et al. [16] have presented an excellent review on the classification of sleep stages using deep learning (DL) methods. This study indicated that the CAP database has not yet been explored for sleep scoring using DL methods, despite the data containing a diverse variety of subjects, signals, sleep disorders and sizes. Boostani et al. [17] have published a review paper on sleep stage classification using various databases and different modalities including PSG and EEG. Zhu et al. [18] presented an automated sleep scoring system using the sleep-EDF database and visibility graphs with graph domain features. They obtained an overall accuracy of 87.5% using support vector machine (SVM) classifier in classifying six sleep stages. Kim et al. [19] used CAP database for sleep stage classification using heart rate variability (HRV) obtained from ECG signals obtained from 13 healthy subjects [20]. In addition, they did not consider a 6-class classification task, but used a binary classification problem. They applied the empirical mode decomposition (EMD) method for noise reduction in (HRV) detrended fluctuation analysis (DFA) and related the noise-reduced fractal property of HRV to the sleep stages of the subjects. Cui et al. [21] used the Institute of Systems and Robotics, University of Coimbra (ISRUC) sleep database and performed 5-class sleep stage classification using EEG, EOG and EMG channels and convolutional neural networks. Sharma et al. [22] performed six-class sleep stage classification by employing a three-band time-frequency localized (TBTFL) wavelet filter bank (FB) approach. They used an EEG channel of 100 Hz sampling rate from the sleep-EDF database and obtained an overall accuracy of 89.5% using SVM classification. However, they have not used a CAP database. Timplalexis et al. [23] carried out 5-class sleep stage classification using a combination of time- and frequency-based features and obtained an overall classification accuracy of 88.88% using an EBT classifier. Tripathi et al. [24] used dispersion entropy and bubble entropy features and a hybrid classifier. They used only 25 subjects among which six were healthy (H), seven were insomniac (Ins), one brux patient, one sleep-disordered breathing (SDB) patient and 10 REM-behaviour disorder (RBD) patients from the CAP database. In addition, they obtained an overall accuracy of 71.68% for 6-class sleep stage classification, which is significantly lesser than the model proposed by us in this study. Recently, Widasari et. al. [25] employed only 51 subjects from the CAP database (16 were healthy, nine were insomniac, four were suffering from SDB and 22 were suffering from RBD) in their study. In addition, they performed only 4-class (W, S1 + S2, S3 + S4, REM) sleep stage classification using sleep quality features and EBT classifier and achieved overall classification accuracy of 86.27%. It is to be noted that all the previous studies have used only unbalanced data and we are the first group to use the balanced dataset in this study. A summary of the state-of-the-art automated sleep stage classification studies conducted is given in Table 1.

In this work we have proposed an automated sleep stage classification system using multi-level wavelet decomposition and norm-based feature extraction, followed by classification using various supervised classifiers. The EEG signals of healthy and sleep disorder patients are fed as input to the automated system to obtain the sleep stages scoring. We have used only one or two EEG channels, hence practical installation is simple and easier as compared to other state-of-the-art techniques, which used PSG [26,27] or several EEG channels and other physiological signals for automated sleep-stage scoring. Subject’s comfort level is also improved as compared to sleep scoring using multi-modal signals.

## 2. Material Used

The EEG dataset used in this study was taken from openly accessible physionet’s CAP sleep database [28,29]. The sleep scoring was done by a team of sleep experts of the Sleep Disorders Centre of the Ospedale Maggiore of Parma, Italy.

The CAP sleep database consists of 108 PSG (polysomnographic) recordings registered at the Sleep Disorders Centre of the Ospedale Maggiore of Parma, Italy. It contains atleast three EEG (Electroencephalogram) channels ( C3 or C4, F3 or F4 and O1 or O2, with reference to A1 or A2), electrooculogram (EOG) channels, submentalis muscle Electromyogram (EMG), respiration signals, bilateral tibial EMG and one electrocardiogram (EKG). The EEG channels additionally includes bipolar channels like F3-C3, Fp1-F3, P3-O1, C3-P3, C4-P4, Fp2-F4, P4-O2 and F4-C4. Among all these channels, two EEG channels namely F4-C4 (bipolar) and C4-A1 (unipolar) are considered for this study as they are present in the maximum number of PSG recordings in this database. The CAP sleep database includes healthy subjects and patients having seven different kinds of sleep disorders such as insomnia, bruxism, narcolepsy, NFLE, PLM, RBD and SDB. The age of subjects varies in the range of 14–82 years, and their average age is around 45 years. A total of 61% of the subjects are men (66 people), and 38% are women (42 people). Most of the studies conducted using the CAP sleep database are on cyclic phase detection [30,31,32,33,34,35]. There are many studies on sleep stage detection using other datasets but there is no study available in literature on sleep stage classification using the CAP sleep database.

Out of 108 subjects, 16 are completely healthy, 40 are diagnosed with nocturnal frontal lobe epilepsy (NFLE), 22 are troubled by REM behaviour disorder, 10 by periodic leg movement (PLM), nine are insomniac, five were narcoleptic, four are facing sleep-disordered breathing and two are diagnosed with bruxism [36]. Sampling frequencies of EEG signals varied between 100 Hz and 512 Hz. A total of 80 subjects with EEG recordings sampled at 512 Hz, as mentioned in Table 2, are considered for this study (48 are male and 32 are female subjects). These subjects are taken on the basis of availability of F4-C4 and C4-A1 EEG channels with sampling frequency of 512 Hz.

The sleep scoring was provided by trained experts, in accordance with the Rechtschaffen and Kales rules [15]. Different stages were annotated as W for wake, S1–S4 for NREM sleep stages and R for REM (Rapid Eye Movement) stage. The details of the total number of epochs of individual stages for all types of patients are shown in Table 3.

### Balancing the Dataset

Intervals corresponding to various sleep stages vary from one person to another person. An average adult’s one-night sleep consists around 2–5% of total time in S1 stage, 45–50% of total time in S2 stage, 5–10% in S3 and S4 stage, and remaining 20–25% time in the REM stage [22,37]. As a result, sleep stage recordings inherently contain unbalanced data and require data balancing in order to yield better models which provide unbiased and robust classification results. In this work, we have also balanced all the data subsets using over-sampling and under-sampling techniques. It can be clearly observed from Table 3 that the number of epochs corresponding to S1 sleep stage constitute only 4.36% of total number of epochs whereas S2 sleep stage constitute around 35.50% of complete dataset. Thus, in order to carry out an unbiased classification, number of epochs corresponding to S1 are increased to 16.67% of total epochs using over-sampling by replacement method. At the same time, epochs corresponding to S2 stage are decreased to 16.67% of total epochs using under-sampling technique. Similarly, all other sleep stages are also brought to 16.67% of total epochs by either over-sampling or under-sampling as per the requirement. Thus, the number of epochs in all six classes are made equal in proportion to perform better classification. The number of epochs used in various classes for different data subsets is shown in Table 4.

## 3. Methodology

**Figure 1:**
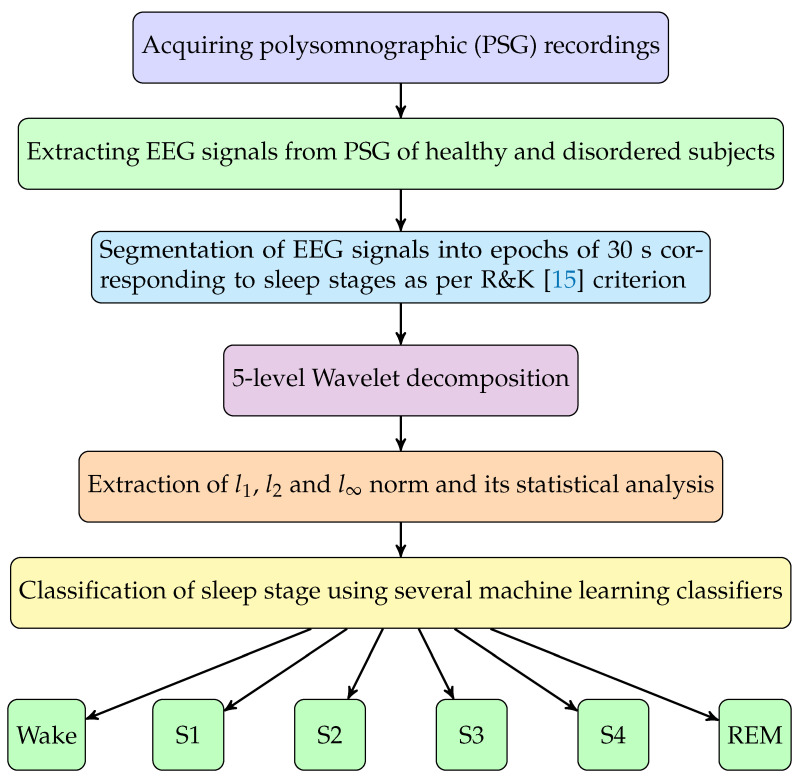
Flowchart of the proposed methodology.

The flow diagram of the proposed method is given in Figure 1. We have explained the data acquired and method used in the following subsections.

### 3.1. Data Acquisition: Acquiring PSG Recordings

As already mentioned in the previous section, data of 80 subjects including six healthy and 74 subjects with sleep disorders were downloaded from physionet’s CAP sleep database. We created a total of 10 data subsets namely ‘healthy’, ‘insomnia’, ‘bruxism’, ‘narcolepsy’, ‘NFLE’, ‘PLM’, ‘RBD’, ‘SDB’, ‘all disordered’ and ‘all subjects combined’. We performed sleep stage classification on each of these data subsets. For each of these data subsets, a matrix containing epochs corresponding to all six sleep stage was formed. The description of these data subset is given below:

- **Healthy**: This data subset contains sleep stage epochs of only healthy subjects with no significant pathology reported. Six healthy subjects are taken in this data subset with a total of 6063 epochs of 30 s duration each.
- **Insomnia**: In this data subset, sleep stage epochs of seven patients suffering from insomnia disorder are taken with a total of 8551 epochs.
- **Bruxism**: This data subset represents 427 epochs obtained from one patient suffering from bruxism sleep disorder.
- **Narcolepsy**: It contains a total of five patients with 5614 epochs of 30 s duration each.
- **NFLE**: This data subset contains 26,883 epochs corresponding to 27 patients suffering from nocturnal frontal lobe epilepsy (NFLE) sleep disorder.
- **PLM**: (Periodic leg movement disorder) This data subset contains 7574 epochs of 30 s duration obtained from 9 PLM patients.
- **RBD**: (Rapid eye movement (REM) behaviour disorder) In this data subset, 22,676 epochs are obtained from 22 RBD patients.
- **SDB**: (Sleep disordered breathing) A total of 2879 epochs of 30 s duration are obtained from three patients with SDB sleep disorder.
- **All Disordered**: This data subset is comprised of 74,574 epochs obtained by combining all 74 disordered subjects.
- **All subjects combined**: This data subset contains 80,667 epochs obtained by combining all types of 80 subjects considered in this study. It includes healthy subjects as well as all disordered subjects.

Each PSG recording contained several types of signals including EEG, EOG, EMG and ECG. We have used two channels F4-C4 and C4-A1 EEG signals individually and jointly.

### 3.2. Segmentation of Sleep Stages into 30 s Epochs

With the help of hypnogram, segregation of different sleep stages present in the EEG recordings was done as per the R & K criteria [15]. Thus, each epoch has been labelled as wake, S1, S2, S3, S4 and REM.

### 3.3. Wavelet Filtering

There are multiple applications of two-channel filter banks (FB) in various fields like analysis of biomedical signals, image processing and communication [22,38,39,40,41,42,43,44]. The FB used in this work is designed in using an Eigenfilter-based approach. The FB is designed by optimizing an objective function and a multiple objective function. The objective function is a convex quadratic function of errors in the pass-band and stop-bands, joint bandwidth duration localization. We used a linear phase optimal biorthogonal wavelet filter bank (OBWFB) in which the analysis filter is a halfband filter. While designing the filter bank, the first step was to design a halfband analysis lowpass filter (HALF) formulating a linearly constrained convex quadratic optimization problem that employed an objective function having a convex combination of passband and stopband errors and bandwidth-duration concentrations of the filter. After designing the half-band analysis filter (HALF), the next step is to design the synthesis lowpass filter (SLPF) in a manner similar to designing HALF but with some variations like (i) avoiding SLPF to be constrained as halfband filters, and (ii) along with the regularity conditions, so that the perfect reconstruction conditions is satisfied.

### 3.4. Wavelet Decomposition

Five level one-dimensional wavelet decomposition of each epoch is done using an optimal biorthogonal wavelet filter as explained above. The five level of decomposition produced six different sub-bands of EEG epochs.

These sub-bands are later used to compute discriminating features.

### 3.5. Extraction of $l_{1}$, $l_{2}$ and $l_{\infty }$ Norm

The discriminating features used for classifying six different classes (W, S1, S2, S3, S4 and REM) are $l_{1}-norm$, $l_{2}-norm$ and $l_{\infty }-norm$. The $l_{m}-norm$ [45] of any discrete-time signal *u*[*n*] is defined as

$${∥u∥}_{m}={\left(\sum_{n=1}^{\infty }{\left|u\left[n\right]\right|}^{m}\right)}^{\frac{1}{m}},m\in Z^{+}$$

In this study, we used m=1 and m=2.

#### $l_{\infty }-norm$ or Peak Absolute Value

The $l_{\infty }-norm$ of a signal gives the maximum absolute value among all the samples of a discrete-time signal. Thus, it is also known as peak absolute value.

$${∥u∥}_{\infty }={\left|u\right|}_{max}$$

Thus, we get a total of 18 features after combining all three norms.

### 3.6. Classification and Validation

Classification of all six stages is performed using all extracted norm-based features. These norm-based features are fed to all the available supervised machine learning classifiers namely decision trees [46,47], logistic regression [48], naive bayes [49], support vector machines (SVM) [46,50], K-nearest neighbours (KNN) [51], ensemble bagged trees (EBT) [52], classifiers and discriminant analysis [53] to select the optimum performing classifier. All the classifiers are developed using a 10-fold cross-validation strategy.

Among all classifiers mentioned above, EBT classifier has yielded optimum performance. EBT classifier is a combination of bagging algorithm and decision tree classifier [52]. In the bagging algorithm, several subsets of data from the training sample are chosen randomly with replacement. Now, each collection of subset data is used to train their decision trees. As a result, we end up with an ensemble of different models and improve classification performance and reduce over-fitting [54]. Averages of all the predictions from different trees are used which is more robust than a single decision tree. Bagging algorithm is used to reduce variance of decision tree.

**Figure 2:**
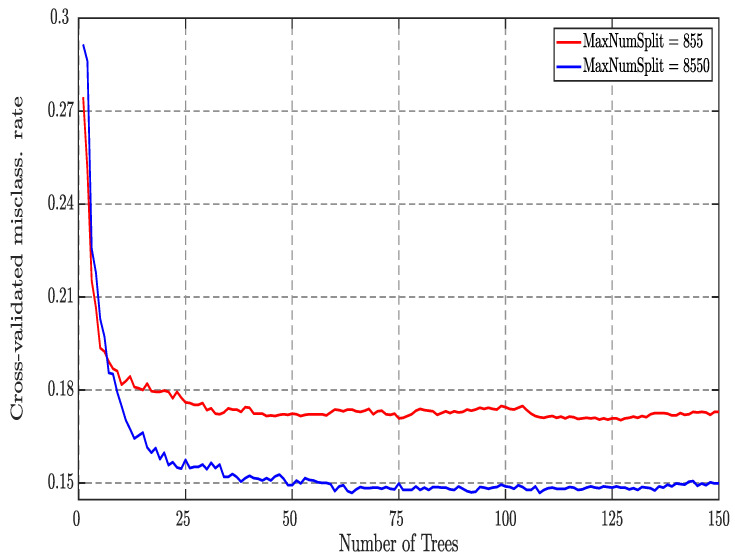
Tuning of hyper parameters of the ensemble of bagged trees (EBT) classifier: Mis-classification error vs. number of trees.

In order to optimize hyper parameters for each of ten classification tasks, we observed the mis-classification error rate by varying the number of splits and maximum number of trees. The maximum number of trees is varied in the range of 10 to 250, number of splits is varied in the range of 1 to n−1, in the steps of 100 from 1 to n−1, (where *n* is the total number of epochs) and then we drew a graph between mis-classification rate versus number of trees (Figure 2). We obtained the converging performance for the split equal to (n−1). The parameters corresponding to minimum error are chosen as the optimal parameters. Figure 2 shows sample EEG plot of tuning of hyper parameters of EBT classifier. The figures also shows the optimal values for number of learners = 65, number of splits = 8550 and learning rate = 1.

## 4. Results

In this work, we have proposed an automated classification of sleep stages using EEG channels. Six stages of sleep, namely: wakefulness (W) stage and five sleep stages namely S1, S2, S3, S4 and REM are classified for normal and abnormal sleep patients. This work was performed on a machine having 8 GB RAM and AMD Ryzen-5 3550 H processor with MATLAB R2020a (version 9.8.0.1323502) installed in it. For classification, we used EEG recordings of six healthy subjects with a total of 6063 epochs (30 s each) and 74 sleep disorder patients which yielded a total of 74,604 epochs (of 30 s each). Thus, a total of 80,667 epochs (of 30 s each) are used for this study. A detailed summary related to number of epochs of different sleep stages and wake (W) of all subjects can be found in Table 3.

We have analysed the features extracted using analysis of variance (ANOVA) technique with Fisher’s least significant difference post-hoc test [55]. The *p*-values obtained from ANOVA test indicate the clinical significance of features. The ranking (of features), *p*-values, mean and standard deviation of all six channels obtained using bipolar (F4-C4) and unipolar channels (C4-A1) are shown in Table 5 and Table 6, respectively. It can be noted from these features that *p*-values corresponding to each feature are almost zero, which indicates that all the features considered in the classification task are statistically significant. As the number of features are only 18 and *p*-values are almost zero, we have not included any features selection algorithm.

**Figure 3:**
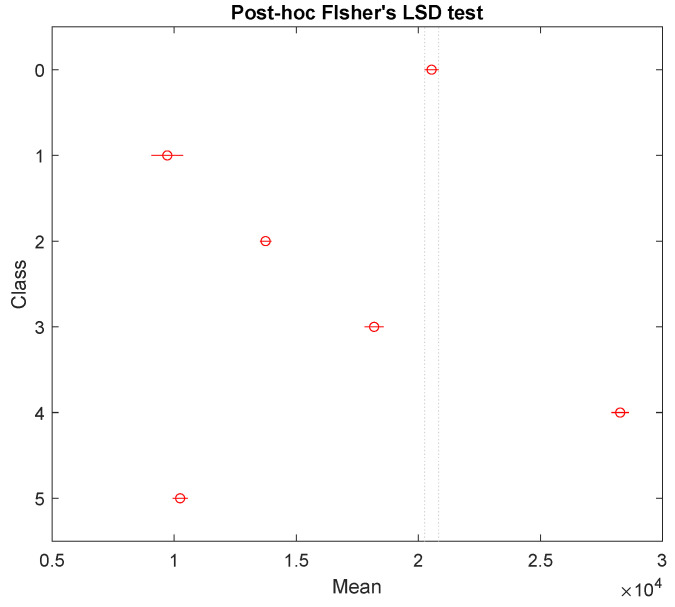
Results of ANOVA post-hoc test obtained using the whole database.

One-way ANOVA [56] shows whether one or more group on which it is performed have any statistical differences based on their means. One-way ANOVA tests the null hypothesis which says that the statistical mean of all groups in consideration is equal. If the one-way ANOVA returns a statistically significant result, then we reject the null hypothesis and accept the alternate hypothesis which says that the statistical mean of all groups in consideration are not equal and hence it provides the evidence of difference. Here, we have used one-way ANOVA with confidence level of 95% and we observe that the null hypothesis is rejected and alternate hypothesis is accepted. However, one-way ANOVA does not signify which particular group is different from the other. Therefore, we used a post-hoc Fisher’s lease significant difference (LSD) [57] test to see which group differs from other and by what margin. We used $l_{1}-norm$ feature of sub-band 1 present in ‘all subjects combined’ dataset and observed the difference in the statistical mean of all six classes as shown in Figure 3.

Sleep recordings inherently contained unbalanced data due to the varying duration of different sleep stages during night sleep. This may result in unequal number of epochs in different sleep stages. As a result, we may get biased and improper sleep stage classification results in favour of the sleep stage having the maximum number of epochs. To avoid this, we have also balanced each data subset using over-sampling (by replacement) and under-sampling techniques. In the over-sampling (by replacement) method, we randomly selected a few epochs from already available epochs and replicated them to increase the epoch count and make it comparable to the epoch count of other classes. Thus, each classification task mentioned above is carried out in two phases as mentioned below:

(i) Classification without balancing the number of epochs among sleep stages.
(ii) Classification after balancing the number of epochs among sleep stages.

We collected sleep data corresponding to both unipolar (C4-A1) and bipolar (F4-C4) EEG channels and performed classification individually as well as after combining both channels. Unipolar EEG channel yielded better classification accuracies as compared to the bipolar channel. However, a combination of both channels (F4-C4 + C4-A1) performed better than individual channels. The classification results are obtained using EBT with 10-fold CV. In 10-fold CV, a complete database is segmented into 10 equal folds and the training is done using nine folds and the remaining one fold is used for testing. Thus, the classification is done in 10 iterations, and then average of 10 folds is considered as the average accuracy. To ensure the robustness of the model, the classification using 10-fold CV is repeated five times for each of 10 datasets and the average of accuracies obtained in each run is then taken as the final measure of classification performance. The details of classification measures corresponding to each of five trials of 10-fold CV and their average are presented in Table 7. Table 8 summarizes the results obtained for all individual data subsets and both EEG channels individually and with the combination of the two channels.

Detailed descriptions corresponding to each classification are given below. In classification task 1, we started with sleep stage classification of only healthy subjects with an unbalanced epoch distribution containing 6063 epochs. It yielded an average classification accuracy of 78.3% using the EBT classifier. The confusion matrix obtained for the classification of healthy subjects is shown in Table 9. The individual classification accuracies obtained for various sleep stages namely: W, S1, S2, S3, S4 and REM are 95.84%, 94.94%, 85.87%, 92.4%, 96.59% and 91.95%, respectively. Generally, in classification problems, the accuracy rate (ACC) is used to compare the performance of studies in this domain, but this metric is accurate if the number of observations is equal to classes. It can be clearly seen in Table 3 that the number of observation among different classes are not equal. Therefoe, ACC is not the best metric to evaluate such a system. Hence, in recent years, Cohen’s Kappa coefficient (κ) is used to evaluate such systems. As a rule of thumb, the value of κ in the range of 0.75 to 1 is considered an excellent classification, κ in between 0.4 and 0.7 is interpreted as fair to good agreement and κ below 0.4 is said to be poor classification agreement [58]. The value of κ = 0.7212 ± 0.0069 is obtained for healthy subjects. The unbalanced healthy data subset had 6063 epochs for S1 and S2 sleep stages with 280 and 2172 epochs, respectively. In order to make the epoch count in all six classes equal, we increased the number of epochs in S1 stage from 280 to 1000 by randomly shuffling and replicating random epochs from the already available 280 epochs of S1 stage. For the same reason, we randomly removed 1172 epochs from the already available 2172 S2 stage epochs to make the epoch count equal to 1000. Similarly, all other stages are also balanced in the same manner. After balancing, the overall classification accuracy is improved from 78.3% to 87.9%. The confusion matrix obtained for the balanced healthy data using the EBT classifier with 10-fold CV can be seen in Table 10. The confusion matrices also included a column corresponding to F1 score for all six classes.

We also have data subsets belonging to seven types of sleep disorders, namely insomnia, bruxism, narcolepsy, NFLE, PLM, RBD and SDB. In CT-2, we started with sleep stage classification of insomniac subjects containing 8551 epochs (30 s) in total. It yielded an average classification accuracy of 85.4% using the EBT classifier. The confusion matrix obtained after classification of insomniac subjects is shown in Table 11. Stages like W, S1, S2, S3, S4 and REM yielded an accuracy of 93.94%, 97.1%, 89.5%, 95.92%, 98.27% and 95.97%, respectively. The value of Cohen’s Kappa coefficient is found to be 0.7867 ± 0.0056, which is in good agreement. After balancing, 8400 epochs out of 8551 are taken from insomnia data subset leading to 1400 epochs in each class. This led to the improvement in the classification accuracy from 85.4% to 92.8%. The confusion matrix corresponding to the balanced insomnia data is shown in Table 12.

In CT-3, we have analysed bruxism sleep disorder subjects. The CAP has only two subjects with bruxism and only one out of two has a sampling frequency of 512 Hz for EEG channel. Hence, we considered only one bruxism subject with 427 sleep stage epochs (30 s) corresponding to bruxism. We obtained an average classification accuracy of 66.7% using the EBT classifier with κ of 0.5578 ± 0.0297. The confusion matrix obtained after classification of bruxism subject is shown in Table 13. The sleep stages of W, S1, S2, S3, S4 and REM yielded an accuracy of 88.06%, 88.99%, 79.86%, 87.82%, 90.4% and 96.49%, respectively. The lower values of overall classification accuracy and κ are due to less number of sleep stage epochs. In bruxism data subset, 426 epochs are considered for balancing which led to the increase in classification accuracy from 66.7% to 82.4%. The confusion matrix for the same can be seen in Table 14.

In CT-4, we analysed five subjects belonging to narcolepsy sleep disorder which yielded a total of 5614 sleep stage epochs with unbalanced epoch distribution among six classes. We obtained an overall classification accuracy of 79.3% using the EBT classifier and κ = 0.7301 ± 0.0070. The confusion matrix obtained after the classification is shown in Table 15. The six sleep stages, namely W, S1, S2, S3, S4, REM yielded an accuracy of 93.73%, 94.58%, 87.41%, 93.82%, 97.27% and 91.5%, respectively. After balancing the epochs, a total number of 5610 sleep epochs are used for classification leading to 935 epochs in each sleep stage. After balancing, its classification accuracy increased from 79.3% to 88.2%. The confusion matrix corresponding to the balanced narcolepsy data is shown in Table 16.

In CT-5, we processed 27 NFLE subjects yielding a total of 26,883 epochs which forms one-third of the complete dataset. We observed an average classification accuracy of 77.5% using the EBT classifier and κ = 0.6914 ± 0.0035 for the unbalanced NFLE data subset. The confusion matrix for the same can be seen in Table 17. Individual stages, namely W, S1, S2, S3, S4, REM yielded an accuracy of 95.16%, 96.29%, 84.05 89.81%, 95.69% and 93.3%, respectively. After balancing, from a total of 26,880 epochs 4480 epochs are considered for each of the six classes. Then the classification accuracy is increased from 77.5% to 86.6%. The confusion matrix for the balanced NFLE data is shown in Table 18.

The CT-6 which includes PLM subjects has obtained an overall classification accuracy of 78.0% using the EBT classifier and κ coefficient = 0.7296 ± 0.0061. The confusion matrix for the same is shown found in Table 19. Sleep stage namely W, S1, S2, S3, S4, REM yielded individual classification accuracy of 94.35%, 95.84%, 86.55%, 90.22%, 95.97% and 95.48%, respectively. For the PLM data subset, the number of epochs in each class is made equal to 1262 and for this balanced dataset, we observed the classification accuracy reaching 85.8% from 78.0%. The confusion matrix for the balanced PLM data classification is shown in Table 20.

In CT-7, all 22 RBD subjects present in the CAPSLPDB are taken for this study yielding a total of 22,676 sleep stage epochs. Overall classification accuracy of 71.9% is obtained using the EBT classifier and κ is found to be 0.6372 ± 0.0039. The confusion matrix for the same is shown in Table 21. Sleep stage namely W, S1, S2, S3, S4, REM yielded individual classification accuracy of 92.26%, 96.3%, 80.89%, 89.88%, 95.65% and 89.23%, respectively. During balancing of epochs in the RBD data subset, the number of epochs in each sleep stage is made equal to 3779. It increased the classification accuracy form 71.9% to 81.0% and the confusion matrix for the same is shown in Table 22.

CT-8, which analysed SDB subjects, obtained an overall classification accuracy of 74.3% using the EBT classifier with κ of 0.6276 ± 0.0117. The confusion matrix for the same is presented in Table 23. Sleep stages W, S1, S2, S3, S4, REM yielded individual classification accuracy of 91.28%, 89.89%, 82.81%, 92.01%, 95.97% and 96.28%, respectively. The balancing of data resulted in 480 epochs in each sleep stage. As a result, we obtained an overall classification accuracy of 86.9% for the balanced data and the confusion matrix is shown in Table 24.

After processing all seven types of disorders individually in previous CTs, we combined them in a single data subset named *all disordered* which is a combination of 74 patients with sleep disorders with 74,604 epochs of 30 s duration each with unbalanced epoch distribution among sleep stages. After classification of this data subset, we observed an overall accuracy of 75.6% using the EBT classifier and κ = 0.6780 ± 0.0021. The confusion matrix for the same classification is shown in Table 25. Individual sleep stages namely W, S1, S2, S3, S4, REM yielded an accuracy of 93.22%, 95.92%, 83.13%, 90.87%, 96.19% and 91.94%, respectively. We have also performed similar balancing on the ’all disordered’ data subset which contained epochs of all seven types of disordered subjects and considered the number of epochs in each of six sleep stages equal to 12,500 with total of 75,000 epochs for all six classes. Balancing operation on this data subset improved the overall classification accuracy to 84.8% from 75.5% for the same classifier. The confusion matrix for the balanced data is shown in Table 26.

Lastly, epochs corresponding to six healthy subjects are also added in the disordered data subset and a new data subset namely *all combined* is formed for CT-10, which contained a total of 80,667 sleep stage epochs corresponding to all types of subjects. For unbalanced data, classification yielded an overall accuracy of 75.5% using the EBT classifier and κ = 0.6697 ± 0.0020. The confusion matrix corresponding to this classification is shown in Table 27. Individual sleep stages, namely W, S1, S2, S3, S4 and REM yielded an accuracy of 93.07%, 95.89%, 82.56%, 90.81%, 96.11% and 91.48%, respectively. While balancing, the number of epochs in each sleep stage is made equal to 14,000 with total of 84,000 epochs for all six stages. The classification of balanced data yielded an accuracy of 85.1% and the confusion matrix for the same is shown in Table 28. Table 29 represents the epoch distribution and obtained accuracies for ‘healthy’, ‘all disordered’ and ‘all combined’ data subsets.

Table 29 and Table 30 quickly summarize the epoch distribution and obtained accuracies for ‘all subjects combined’, ‘healthy’ and ‘all disordered’ datasets. All the accuracies mentioned below are obtained from the EBT classifier.

**Figure 4:**
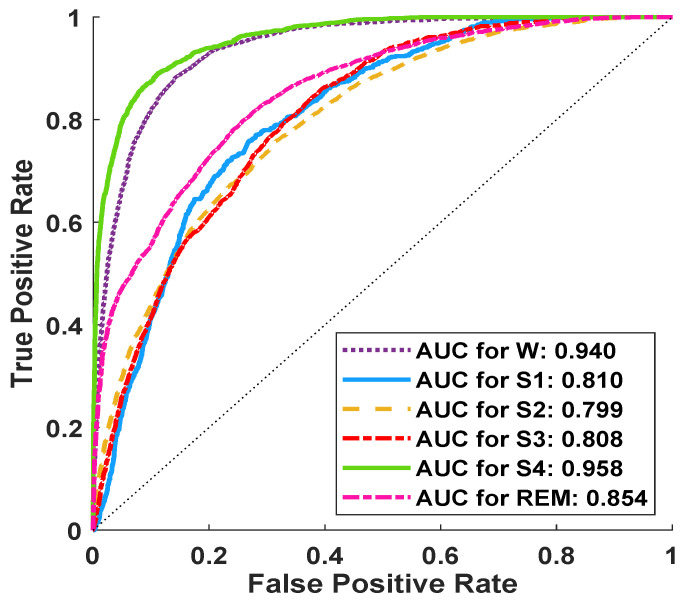
ROC curves obtained for the classifier whole database using the EBT with 10-fold CV.

The receiver operating characteristic (ROC) curves are generally plotted for the binary classification problem. Since, the classification in this work involves six-class classification tasks, we have drawn ROC taking one class at a time as positive class and the remaining five classes as negative class. In Figure 4, we have shown the ROC plot corresponding to the whole database. From the figure it is clear that the area under the curve varies between 0.80 to 0.94 which indicates the effectiveness of the proposed model in discriminating the sleep stages.

## 5. Discussion

The results obtained by our study indicate that the proposed model achieved high classification performance using EEG signals with unbalanced and balanced sleep datasets. This study is the first to attempt 6-class sleep stage classification using single/dual channel EEG signals with high sampling frequency of 512 Hz. In addition, this is the first study to consider the sleep stages of different sleep disorders such as insomnia, bruxism, narcolepsy, NFLE, PLM, RBD and SDB. In this work, we presented the results of unipolar and bipolar EEG channels individually as well as combined. The EEG epochs are decomposed into sub-bands using a new class of optimized wavelet filters. Different optimal wavelet filters of varying lengths and vanishing moments with different levels of decomposition have been used to obtain optimum performance. Two different EEG channels, namely F4-C4 and C4-A1, along with their combination have been used to obtain deeper insights. Wavelet-based norm features of six sub-bands have been computed and fed to various classifiers including EBT to choose the optimum performing classifier.

The PSG is considered the gold standard to score sleep stages and diagnose sleep disorders. The PSG-based techniques require multiple wired sensors to record the activities of multiple physiological signals (such as EEG, EMG, ECG, EOG, respiratory signals) and time-consuming analysis procedures. Moreover, the sleep recordings need to be conducted overnight in a specialized sleep laboratory or hospital. Further, sometimes PSG recording process may cause inconvenience to older people who often suffer from sleep disorders. Hence, it is desirable to explore some new techniques and methods that can produce accurate results similar to manual sleep staging PSG-based methods, which are simple, less time-consuming, inexpensive and convenient to the patients. Our proposed study is a humble attempt in that direction which needs to be tested independently with more diverse and bigger databases or cohorts.

We perform the wavelet processing and feature extraction using single channel or dual channel EEG epochs of duration 30 s only (instead of 1 min or higher duration). Due to this simplification, the proposed method has low computational cost and therefore it that can be implemented in an embedded hardware device.

We have considered the classification of six classes, whereas other studies [19,25] considered two class or three-class classification tasks. The most challenging task is classification of subclasses of NREM (i.e., S1–S4). The classification of S1–S4 is missing in the existing literature but we got very good accuracy for the classification of subclasses of NREM also. The most challenging task in sleep stage classification is to score S1 stages accurately and we have attained reasonable accuracy in identifying the S1 stage along with S2–S3.

Novelty of the work is that the model developed can be used for sleep-scoring of not only good sleeper but can also be used for the subjects suffering from various sleep disorders in automated speedy fashion without much complexities. There are seldom any study which focus on sleep-scoring of sleep disorder subjects. Since in the CAP data mostly subjects are elderly, the model can be further used and tested for identifying sleep disorders in the elderly. An accurate sleep-stage scoring of sleep disordered patients will help in diagnosis and prognosis of disorders, which is much needed and highly desirable for elderly persons.

We have used a novel optimal filter bank. We have used linear phase optimal biorthogonal wavelet filter banks (OBWFB) in which the analysis filter is a halfband filter [59]. The halfband pair filter bank (HPFB) design technique introduced by Phoong et al. [60] and its other variants [61] are all indirect approaches for filter design. These technique have restrictions like lack of control on frequency responses, joint bandwidth-duration localization and control between frequency selectivity and smoothness of filters [62,63,64]. In order to overcome these limitations, we used a filter which is designed using a direct, time-domain approach that avoids the need for the design of intermediate kernels. Unlike the Phoong et al. and Tay et al. [60,61] HPFB technique, the design technique of our linear phase optimal biorthogonal wavelet filter bank is simple and efficient to control the smoothness, frequency selectivity and joint bandwidth-duration localization of filters [65,66,67]. It is to be noted that the analysis filter of the wavelet filter bank used is a half band filter in which half of the filter coefficients are zero; hence, the computational cost of finding sub-bands using the proposed wavelet filter is exactly half of the standard Daubechies wavelet used in the literature [42,68,68]. Moreover, in designing the (HALF), we need not design intermediate kernels unlike other methods [60,61]. Thus, our design method has lower design complexity also.

The notable aspect of this work is that the publicly available CAP sleep database containing normal and seven different types of sleep disorders are used for the first time to develop an automated sleep scoring system. In this work, we have also balanced all the data subsets using over-sampling and under-sampling techniques. In this work, epochs corresponding to minority classes (example: S1) are increased by oversampling to make them proportion to other classes. At the same time, epochs corresponding to classes having large portion of total distribution are brought down by under-sampling. Thus, epochs in all six classes are made equal in proportion to make efficient classification. To develop a robust model and avoid possible over fitting, we have also balanced the data as original data are unbalanced and we obtained the results using both balanced and unbalanced data. To the best of our knowledge, this is the first study to use the balanced CAP database.

It is observed that the unipolar channel C4-A1 performed better for the classification of healthy as well as all seven disordered classes. Thus, we can conclude that single channel (unipolar channel) performed better than bipolar channel. When both the channels are combined then the performance of classification has improved. Since we used only single/dual channel EEG signals instead of complex multichannel multimodal PSG recording, the system complexity is low. Further, we employed EEG epochs of duration 30s only (instead of 1 min or higher duration). Hence, the proposed method has a low computational cost and therefore it can be implemented in an embedded hardware device.

The key features of this study are as follows:

1. To the best of our knowledge, this is the first study to use the whole CAP database that includes 80 subjects with seven different sleep disorders (insomnia, bruxism, narcolepsy, NFLE, PLM, REM, RDB and SBD as well as normal subjects). We have used the highest number of epochs (80,667) in this study which is larger than most of the existing studies. In the existing literature [19,21,25], studies have used only a few healthy subjects.
2. A simple, fast and accurate automated sleep stage detection system is developed.
3. The proposed model has attained high classification performance for all 10 classification tasks considered in this study.
4. To the best of our knowledge this is the first study to perform sleep stage classification of all sleep disorders using the CAP sleep database.
5. As compared to previous studies on sleep stage classification, we have used more data containing sleep stage recordings of 80 subjects with 80,667 sleep stage epochs of 30 s duration each.
6. This is the first study to employ machine learning coupled with optimal wavelet filter bank for sleep scoring detection using EEG signals with a high sampling frequency of 512 Hz.
7. We have employed a new class of optimal wavelet filters to extract the norm-based features of EEG channels.
8. We have used only two EEG channels and extracted norm-based features which makes it simpler and computationally efficient.

The limitations of this are as follows:

- Placement of EEG electrodes on the human skull is a complex task and sometimes it may even cause discomfort to the subjects. Hence, in this work, we have used only two electrodes.
- We used the EEG signals of subjects sampled at 512 Hz sampling frequency, hence we had to eliminate the use of the other 28 subjects (not sampled at 512 Hz). Therefore, we finally used only 80 subjects for this study.
- We obtained the least classification accuracy for S1 sleep stage as the number of data available are minimum using the unbalanced database. However, the performance accuracy of S1 sleep stage is comparable with other sleep stages using balanced database.
- Computation of wavelet-based features takes more time than the ordinary statistical features. However, the same wavelet filter also helps to remove the noise.
- The CAP database has been sleep scored according to the R & K criterion, in which sleep is classified into six stages. Therefore, in the proposed study, we have considered the six-class classification task. However, as per the American Academy of Sleep Medicine (AASM) guidelines for sleep scoring, stages S3 and S4 are combined into a single stage called N3. Thus, as per AASM guidelines, whole-night sleep is divided into five sleep stages: wakefulness (W), N1, N2, N3, and REM instead of six stages as defined by the R & K criterion. This limitation can be overcome by combining stages S3 and S4 into the new stage N3 and presenting the results as per AASM guidelines and not according to R & K rules.

The proposed model achieved high Cohen’s Kappa coefficient values (more than 0.65) for both unbalanced and balanced sleep datasets. In the future, we intend to evaluate the performance of our model with more sleep EEG data and install the developed model in the cloud to get accurate diagnosis of the type of sleep disorder immediately.

## 6. Conclusions

In this work, we have proposed an automated sleep stage classification system using two EEG channels: unipolar (C4-A1) and bipolar (F4-C4). We have used EEG signal recordings of 80 subjects consisting of six healthy subjects and 74 patients suffering from any one of seven sleep disorders, namely insomnia, bruxism, narcolepsy, NFLE, PLM, RBD and SDB. After segmenting each EEG signal into multiple 30 s epochs corresponding to six different classes (wake, S1, S2, S2, S4, REM), 5-level 1-D wavelet decomposition of each epoch is done. Then the norm-based features are extracted from each EEG channel. The duration of various sleep stages vary resulting in unbalanced data with unequal EEG epochs in six classes. In order to avoid bias and obtain better classification results, we performed data balancing using over-sampling and under-sampling techniques and obtained balanced data with almost equal epoch distribution among all six classes.

Our proposed method attained the maximum accuracy of 75.6% and Cohen’s Kappa coefficient of 0.6780±0.0021 for unbalanced data while 85.1% accuracy with Cohen’s Kappa coefficient of 0.8214 for balanced data using the EBT classifier with ten-fold cross-validation strategy. The classification performance of the proposed model indicates that it can reliably classify the sleep stages using 30-s duration with single or dual channel EEG instead of using multichannel multimodal PSG.

As this method is based on only two EEG channels, practical setup is also easier to implement. It helps the sleep experts to devote more time and effort on sleep scoring. In the future, we intend the evaluate the performance of our developed model with more sleep EEG data and use it as cloud-based AI system to detect the sleep disorders immediately. In the future, we intend to extend our study on the CAP database to classify different sleep disorders such as insomnia and narcolepsy using EEG and ECG signals. We also plan to use our developed model for automated sleep stage classification using EOG and HRV signals.

## Figures and Tables

**Table 1:** Summary of the state-of-the-art automated sleep stage classification studies conducted.

Work	Description	Performance (%)
Kim et al. [19] (2017)	**Database:** CAP **Subjects:** 13 Healthy **Signals:** ECG/HRV **Classification:** 2-class [(a)W vs. Sleep; and (b) (S1 + S2) vs. (S3 + S4)] **Features:** Detrended fluctuation analysis(DFA) alpha 1 **Classifier:** k-fold cross validation (k = 13)	**Accuracy:** (a): 73.6% (b): 72.3
Sharma et al. [22] (2018)	**Database:** Sleep EDF **Subjects:** 98 (Healthy) **Signals:** EEG **Channel:** Fpz-Cz, pz-Oz,C4-A1 **Classification:** 6-class [W vs. S1 vs. S2 vs. S3 vs. S4 vs. REM] **Features:** Log energy, Signal fractal dimension, Signal sample entropy **Classifier:** SVM	**Accuracy:** 91.5
Timplalexis et al. [23] (2019)	**Database:** Sleep EDF **Subjects:** 197 Healthy **Signals:** EEG **Channel:** Fpz-Cz, Pz-Oz **Classification:** 5-class [W vs. N1 vs. N2 vs. N3 vs. REM] **Features:** Mixture of time and frequency domain features **Classifier:** EBT	**Accuracy:** 88.88
Tripathi et al. [24] (2020)	**Database:** CAP **Subjects:** 25 (6-H,7-Ins,1-Brux,1-SDB,10-RBD) **Signals:** EEGs **Channel:** F4-C4, C4-P4, P4-O2, C4-A1 **Classification:** 6-class (W vs. S1 vs. S2 vs. S3 vs. S4 vs. REM) **Features:** Dispersion entropy and Bubble entropy **Classifier:** Hybrid classifier	**Accuracy:** 71.68
Widasari et al. [25] (2020)	**Database:** CAP **Subjects:** 51 [16-H, 9-ins, 4-SDB, 22-RBD] **Signals:** ECG **Classification:** 4-class [W vs. (S1 + S2) vs. (S3 + S4) vs. REM] **Features:** Sleep quality **Classifier:** EBT	**Accuracy:** 86.27

**Table 2:** Description of the cyclic alternating pattern (CAP) sleep database.

Subject Type	Subjects Available	Recordings Available @512 Hz	Number-of Epochs	Male & Female	Age (in yrs) (Mean ± Std)
Healthy	16	6	6063	2 M & 4 F	32 ± 4.89
Insomnia	9	7	8551	3 M & 5 F	61.75 ± 10.20
Bruxism	2	1	427	1 M	34
Narcolepsy	5	5	5614	2 M & 3 F	31.6 ± 10.32
NFLE	40	27	26,883	13 M & 14 F	30.03 ± 10.53
PLM	10	9	7574	6 M & 3 F	54.44 ± 6.37
RBD	22	22	22,676	19 M & 3 F	70.72 ± 6.23
SBD	4	3	2879	3 M	69.33 ± 6.12
Total	108	80	80667	48M & 32F	48.25 ± 19.69

**Table 3:** Sleep stage-wise and subject-wise details of epoch distribution in the original unbalanced database.

Sleep Stage	Healthy	Seven Different Disorders							Total	
		Insomnia	Bruxism	Narcolepsy	NFLE	PLM	RBD	SBD	Epochs	(in %)
**Wake**	445	3801	44	1303	3155	1332	5266	495	15,841	19.64%
**S1**	280	223	34	301	1098	266	1048	269	3519	4.36%
**S2**	2172	2456	144	1708	10,630	2748	7446	1324	28,628	35.50%
**S3**	573	670	39	476	2987	955	2880	224	8804	10.92%
**S4**	1184	415	99	568	4108	956	2506	352	10,188	12.63%
**REM**	1409	986	67	1258	4905	1317	3530	215	13,687	16.97%
**Total**	6063	8551	427	5614	26,883	7574	22,676	2879	80,667	

**Table 4:** Sleep stage-wise and subject-wise details of epoch distribution in the created balanced data.

Sleep Stage	Healthy	Seven Different Disorders							Total	
		Insomnia	Bruxism	Narcolepsy	NFLE	PLM	RBD	SBD	Epochs	(in %)
**Wake**	1000	1400	71	935	4480	1262	3779	480	13,407	16.67%
**S1**	1000	1400	71	935	4480	1262	3779	480	13,407	16.67%
**S2**	1000	1400	71	935	4480	1262	3779	480	13,407	16.67%
**S3**	1000	1400	71	935	4480	1262	3779	480	13,407	16.67%
**S4**	1000	1400	71	935	4480	1262	3779	480	13,407	16.67%
**REM**	1000	1400	71	935	4480	1262	3779	480	13,407	16.67%
**Total**	6000	8400	426	5610	26,880	7572	22,674	2880	80,442	

**Table 5:** Statistical analysis using ANOVA for F4-C4 channel.

Feature	Sub-Band	Rank	*p*-Value	W (Mean ± Std)	S1 (Mean ± std)	S2 (Mean ± Std)	S3 (Mean ± Std)	S4 (Mean ± Std)	REM (Mean ± Std)
$l_{1}-norm$	Sb-1	2	0	20,656.14 ± 54,855.48	9281.65 ± 9331.55	13,761.47 ± 9066.79	18,321.05 ± 11,585.59	28,405.90 ± 16,908.07	9989.34 ± 6288.26
	Sb-2	1	0	749.99 ± 1307.83	296.04 ± 692.05	197.74 ± 328.99	162.27 ± 216.34	154.04 ± 354.95	167.73 ± 114.87
	Sb-3	16	0	2740.34 ± 4746.11	877.57 ± 1053.98	644.24 ± 718.22	528.03 ± 452.74	486.95 ± 602.20	560.29 ± 450.23
	Sb-4	13	0	5857.94 ± 9138.12	2190.28 ± 1620.46	1749.38 ± 1380.68	1449.88 ±928.23	1277.17 ± 800.73	1796.56 ±1233.18
	Sb-5	14	0	7843.60 ± 10,155.93	3975.20 ± 2416.01	3764.24 ± 2709.47	3173.00 ± 1925.92	2813.16 ± 1394.38	3579.89 ± 2530.63
	Sb-6	4	0	6880.60 ± 9721.22	4182.79 ± 2983.74	4832.37 ± 3530.33	5016.90 ± 3619.34	5092.04 ± 3125.72	3765.75 ± 2241.55
$l_{2}-norm$	Sb-1	3	0	1368.82 ± 3627.26	624.28 ± 896.76	884.61 ± 663.45	1110.23 ± 718.85	1658.00 ± 980.41	610.15 ± 488.75
	Sb-2	7	1.13 × 10^−121^	16.32 ± 62.57	15.08 ± 130.57	5.61 ± 61.93	4.02 ± 47.44	4.37 ± 57.16	2.85 ± 4.35
	Sb-3	15	0	74.06 ± 136.78	28.04 ± 80.17	17.09 ± 48.82	12.85 ± 35.01	12.22 ± 48.50	14.02 ± 28.81
	Sb-4	11	0	204.88 ± 351.47	76.59 ± 97.83	56.15 ± 65.52	45.08 ± 64.96	39.13 ± 51.53	55.86 ± 48.13
	Sb-5	8	0	363.07 ± 571.78	181.13 ± 167.77	165.59 ± 132.54	138.25 ± 116.83	122.02 ± 81.54	152.98 ± 114.36
	Sb-6	10	0	440.48 ± 803.03	267.51 ± 282.30	298.63 ± 234.43	302.81 ± 239.25	301.61 ± 193.20	225.46 ± 137.81
$l_{\infty }-norm$	Sb-1	9	0	279.62 ± 609.45	139.41 ± 295.40	198.69 ± 189.74	218.39 ± 172.16	287.81 ± 192.23	117.48 ± 135.51
	Sb-2	6	3.39 × 10^−34^	3.01 ± 31.62	6.05 ± 69.16	1.64 ± 34.01	1.16 ± 28.38	1.33 ± 30.19	0.30 ± 0.92
	Sb-3	17	0	11.51 ± 28.84	5.93 ± 37.01	2.69 ± 20.83	1.78 ± 13.42	1.85 ± 20.98	1.89 ± 5.85
	Sb-4	5	0	35.35 ± 76.92	13.10 ± 39.59	7.90 ± 23.04	5.76 ± 20.35	4.83 ± 21.26	7.46 ± 13.76
	Sb-5	12	0	71.53 ± 157.27	33.31 ± 74.26	27.44 ± 42.13	22.45 ± 39.66	19.76 ± 29.94	24.59 ± 33.29
	Sb-6	18	0	97.71 ± 247.88	58.77 ± 126.53	62.01 ± 68.44	58.84 ± 75.50	54.32 ± 47.08	43.76 ± 37.97

**Table 6:** Statistical analysis using ANOVA for C4-A1 channel.

Feature	Sub-Band	Rank	*p*-Value	W (Mean ± Std)	S1 (Mean ± std)	S2 (Mean ± Std)	S3 (Mean ± Std)	S4 (Mean ± Std)	REM (Mean ± Std)
$l_{1}-norm$	Sb-1	1	0	36,981.35 ± 58,883.51	23,963.81 ± 20,242.16	32,003.79 ± 18,531.48	41,632.86 ± 16,256.33	63,699.18 ± 27,718.67	25,232.93 ± 13,663.27
	Sb-2	14	0	1563.97 ± 1513.03	852.80 ± 980.33	534.78 ± 650.33	437.19 ± 466.81	345.76 ± 449.87	358.28 ± 371.59
	Sb-3	5	0	5904.82 ± 5616.39	2981.21 ± 2803.20	1990.35 ± 2449.27	1665.68 ± 1614.53	1349.09 ± 1264.19	1361.41 ± 1437.16
	Sb-4	13	0	12,578.84 ± 11,387.83	6533.51 ± 4718.18	4690.19 ± 3691.48	4069.94 ± 2870.76	3403.56 ± 2202.58	4172.80 ± 2942.39
	Sb-5	3	0	15,775.64 ± 12,303.67	10,209.32 ± 4514.26	8824.32 ± 4143.84	7721.53 ± 2683.56	6881.04 ± 2166.72	7998.54 ± 3353.39
	Sb-6	6	0	14,728.59 ± 11,745.06	11,078.86 ± 5189.41	11,424.13 ± 4806.48	11,800.24 ± 3853.04	11,895.95 ± 4454.35	9165.82 ± 3029.86
$l_{2}-norm$	Sb-1	4	0	2425.35 ± 3989.36	1578.19 ± 1751.27	2019.55 ± 1399.18	2501.36 ± 1138.42	3702.32 ± 1584.33	1554.12 ± 1183.25
	Sb-2	7	0	30.16 ± 64.44	24.86 ± 126.99	11.47 ± 62.72	8.30 ± 46.92	7.33 ± 57.06	6.42 ± 8.19
	Sb-3	16	0	150.45 ± 159.77	81.05 ± 106.23	49.31 ± 73.79	37.86 ± 50.84	30.66 ± 53.66	34.96 ± 47.16
	Sb-4	2	0	431.08 ± 428.95	228.16 ± 210.81	153.65 ± 150.42	125.85 ± 119.98	103.22 ± 83.31	138.40 ± 126.72
	Sb-5	17	0	719.14 ± 662.50	463.36 ± 263.17	391.92 ± 214.74	337.04 ± 164.01	299.96 ± 111.59	349.51 ± 177.53
	Sb-6	15	0	904.04 ± 887.55	681.53 ± 364.61	695.50 ± 313.77	702.02 ± 251.02	697.05 ± 262.48	544.97 ± 195.84
$l_{\infty }-norm$	Sb-1	18	0	491.43 ± 757.98	336.28 ± 510.28	431.10 ± 370.31	477.72 ± 303.09	630.99 ± 313.80	302.60 ± 355.54
	Sb-2	10	3.27 × 10${}^{-39}$	4.30 ± 31.83	7.18 ± 71.13	2.19 ± 33.77	1.39 ± 25.34	1.59 ± 29.97	0.78 ± 1.43
	Sb-3	8	0	20.73 ± 32.27	12.47 ± 38.15	6.43 ± 20.30	4.29 ± 15.56	3.73 ± 23.68	5.22 ± 9.53
	Sb-4	11	0	69.69 ± 91.72	37.35 ± 59.70	22.08 ± 39.13	15.64 ± 30.68	11.96 ± 24.28	21.98 ± 34.33
	Sb-5	9	0	136.69 ± 180.07	84.12 ± 97.30	66.58 ± 67.29	54.39 ± 55.85	47.25 ± 37.82	61.45 ± 68.00
	Sb-6	12	0	183.10 ± 263.80	137.42 ± 131.19	138.07 ± 96.04	129.94 ± 79.24	120.41 ± 61.44	105.42 ± 74.07

**Table 7:** Summary of classification average accuracy obtained during each trial using the EBT classifier with 10-fold cross validation (CV).

Type of Subject	Channel	Trial 1	Trial 2	Trial 3	Trial 4	Trial 5	Mean ± Std
Healthy	F4-C4	74.96	71.86	72.46	71.56	73.66	72.9 ± 1.26
	C4-A1	73.1	72.2	74.8	73.4	72.5	73.2 ± 0.91
	F4-C4 + C4-A1	76.26	77.66	79.06	79.66	78.86	78.3 ± 1.21
Insomnia	F4-C4	86.74	86.14	86.14	85.64	85.84	86.1 ± 0.37
	C4-A1	86.76	85.46	85.46	86.56	85.26	85.9 ± 0.63
	F4-C4 + C4-A1	85.14	86.24	84.44	84.54	86.64	85.4 ± 0.89
Bruxism	F4-C4	62.34	64.84	63.74	63.94	62.64	63.5 ± 0.91
	C4-A1	66.34	64.04	63.94	66.24	65.94	65.3 ± 1.08
	F4-C4 + C4-A1	67.78	65.78	65.48	68.58	65.88	66.7 ± 1.24
Narcolepsy	F4-C4	77.06	78.46	76.16	77.16	76.16	77 ± 0.85
	C4-A1	76.52	78.02	74.32	76.22	75.42	76.1 ± 1.23
	F4-C4 + C4-A1	80.2	77.9	77.9	79.8	80.7	79.3 ± 1.18
NFLE	F4-C4	71.8	72.8	72.6	71.1	70.7	71.8 ± 0.82
	C4-A1	72.18	71.98	71.78	72.58	73.98	72.5 ± 0.79
	F4-C4 + C4-A1	76.5	78.5	77.7	78.8	76	77.5 ± 1.09
PLM	F4-C4	73.88	71.58	74.28	72.08	74.68	73.3 ± 1.24
	C4-A1	74.7	74.6	74.9	76.5	73.8	74.9 ± 0.88
	F4-C4 + C4-A1	77.24	78.34	78.14	79.34	76.94	78 ± 0.85
RBD	F4-C4	66.06	67.66	65.76	64.56	64.96	65.8 ± 1.07
	C4-A1	68.56	66.86	66.06	67.76	66.76	67.2 ± 0.87
	F4-C4 + C4-A1	71.38	72.88	70.38	72.78	72.08	71.9 ± 0.93
SDB	F4-C4	70.52	68.12	68.12	69.72	68.02	68.9 ± 1.03
	C4-A1	70.94	72.34	74.24	71.54	73.44	72.5 ± 1.21
	F4-C4 + C4-A1	74.06	74.96	73.96	75.36	73.16	74.3 ± 0.78
Healthy + Unhealthy subjects	F4-C4	70.1	68.6	68.4	71	69.9	69.6 ± 0.97
	C4-A1	68.58	71.78	70.18	70.28	70.68	70.3 ± 1.03
	F4-C4 + C4-A1	73.38	75.78	76.08	76.28	75.98	75.5 ± 1.07

**Table 8:** Performance of sleep stage classification obtained using the unbalanced dataset and EBT classifier with 10-fold CV.

Type of Subject	Total Subjects Available	Recordings Available @512 Hz	Channel	Accuracy (%)	No. of Epochs	Prediction Speed (obs/s)	Training Time (s)
Healthy	16	6	F4-C4	72.9	6063	15,000	25.23
			C4-A1	73.2		17,000	20.25
			F4-C4 + C4-A1	78.3		17,000	22.26
Insomnia	9	7	F4-C4	86.1	8551	23,000	22.73
			C4-A1	85.9		23,000	22.46
			F4-C4 + C4-A1	85.4		13,000	28.69
Bruxism	2	1	F4-C4	63.5	427	1500	4.84
			C4-A1	65.3		1400	4.88
			F4-C4 + C4-A1	66.7		1600	5.02
Narcolepsy	5	5	F4-C4	77.0	5614	11,000	14.00
			C4-A1	76.1		11,000	14.45
			F4-C4 + C4-A1	79.3		13000	15.77
NFLE	40	27	F4-C4	71.8	26,883	21,000	62.38
			C4-A1	72.5		19,000	63.17
			F4-C4 + C4-A1	77.5		20,000	78.32
PLM	10	9	F4-C4	73.3	7574	14,000	18.74
			C4-A1	74.9		9700	27.70
			F4-C4 + C4-A1	78.0		16,000	20.94
RBD	22	22	F4-C4	65.8	22,676	15,000	63.79
			C4-A1	67.2		18,000	54.45
			F4-C4 + C4-A1	71.9		12,000	119.48
SDB	4	3	F4-C4	68.9	2879	5900	10.72
			C4-A1	72.5		5300	12.45
			F4-C4 + C4-A1	74.3		6100	13.53
Healthy + Unhealthy subjects	108	80	F4-C4	69.6	80,667	18,000	239.74
			C4-A1	70.3		18,000	239.80
			F4-C4 + C4-A1	75.5		19,000	286.92

**Table 9:** Confusion matrix corresponding to sleep stage classification of healthy subjects with unbalanced data using the EBT classifier with 10-fold CV.

CT-1: Healthy (Unbalanced Data)							
True Class	Predicted Class						F1 Score
	Wake	S1	S2	S3	S4	R	
Wake	68.8%	15.7%	5.4%	0.9%	0.4%	8.8%	0.71
S1	21.4%	42.5%	23.2%	0.0%	0.7%	12.1%	0.44
S2	1.3%	2.3%	80.9%	6.0%	1.2%	8.4%	0.80
S3	0.9%	0.2%	28.6%	56.9%	12.6%	0.9%	0.59
S4	0.3%	0.1%	1.9%	5.7%	91.7%	0.2%	0.91
REM	1.1%	1.8%	11.8%	0.9%	0.5%	84.0%	0.83
**Cohen’s kappa (±kappa error) = 0.7212 ± 0.0069**							
**Overall Accuracy = 78.3%**							

**Table 10:** Confusion matrix corresponding to sleep stage classification of healthy subjects with balanced data using the EBT classifier with 10-fold CV.

CT-1: Healthy (Balanced Data)							
True Class	Predicted Class						F1 Score
	Wake	S1	S2	S3	S4	R	
Wake	97.7%	1.4%	0.5%	0.1%	0.1%	0.2%	0.96
S1	0.5%	99.3%	0.0%	0.0%	0.0%	0.2%	0.95
S2	2.3%	5.0%	64.1%	12.5%	2.3%	13.8%	0.73
S3	0.3%	0.1%	3.3%	94.0%	2.3%	0.0%	0.86
S4	0.2%	0.1%	1.4%	9.8%	88.5%	0.0%	0.92
REM	2.1%	5.0%	8.0%	1.5%	0.9%	82.5%	0.83
**Cohen’s kappa (±kappa error) = 0.8554 ± 0.0050**							
**Overall Accuracy = 87.9%**							

**Table 11:** Confusion matrix corresponding to sleep stage classification of insomnia patients with balanced data using the EBT classifier with 10-fold CV.

CT-2: Insomnia (Unbalanced Data)							
True Class	Predicted Class						F1 Score
	Wake	S1	S2	S3	S4	R	
Wake	96.2%	0.7%	2.6%	0.1%	0.1%	0.4%	0.93
S1	44.8%	7.2%	37.7%	0.4%	0.0%	9.9%	0.11
S2	8.9%	0.6%	81.6%	3.6%	0.4%	4.9%	0.82
S3	1.3%	0.0%	17.9%	56.9%	9.7%	0.1%	0.73
S4	0.0%	0.0%	2.2%	14.5%	83.4%	0.0%	0.82
REM	4.7%	0.1%	13.8%	0.1%	0.1%	81.2%	0.82
**Cohen’s kappa (±kappa error) = 0.7867 ± 0.0056**							
**Overall Accuracy = 85.4%**							

**Table 12:** Confusion Matrix corresponding to sleep stage classification of insomnia patients with unbalanced data using the EBT classifier with 10-fold CV.

CT-2: Insomnia (Balanced Data)							
True Class	Predicted Class						F1 Score
	Wake	S1	S2	S3	S4	R	
Wake	92.1%	4.0%	2.7%	0.2%	0.3%	0.6%	0.93
S1	0.0%	100%	0.0%	0.0%	0.0%	0.0%	0.95
S2	5.6%	4.5%	74.1%	7.1%	0.7%	7.9%	0.82
S3	0.3%	0.0%	1.1%	97.1%	1.4%	0.0%	0.95
S4	0.0%	0.0%	0.0%	0.0%	100.0%	0.0%	0.99
REM	1.1%	1.1%	3.9%	0.1%	0.1%	93.6%	0.93
**Cohen’s kappa (±kappa error) = 0.9145 ± 0.0034**							
**Overall Accuracy = 92.8%**							

**Table 13:** Confusion matrix corresponding to sleep stage classification of bruxism patients with unbalanced data using the EBT classifier with 10-fold CV.

CT-3: Bruxism (Unbalanced Data)							
True Class	Predicted Class						F1 Score
	Wake	S1	S2	S3	S4	R	
Wake	54.5%	15.9%	18.2%	6.8%	2.3%	2.3%	0.48
S1	41.2%	17.6%	29.4%	0.0%	8.8%	2.9%	0.20
S2	6.9%	5.6%	75.0%	6.3%	2.8%	3.5%	0.72
S3	7.7%	0.0%	46.2%	10.3%	35.9%	0.0%	0.13
S4	4.0%	4.0%	6.1%	5.1%	80.8%	0.0%	0.80
REM	0.0%	0.0%	11.9%	0.0%	0.0%	88.1%	0.89
**Cohen’s kappa (±kappa error) = 0.5578 ± 0.0297**							
**Overall Accuracy = 66.7%**							

**Table 14:** Confusion matrix corresponding to sleep stage classification of bruxism patients with balanced data using the EBT classifier with 10-fold CV.

CT-3: Bruxism (Balanced Data)							
True Class	Predicted Class						F1 Score
	Wake	S1	S2	S3	S4	R	
Wake	90.1%	8.5%	0.0%	0.0%	0.0%	1.4%	0.89
S1	0.0%	97.2%	0.0%	0.0%	2.8%	0.0%	0.82
S2	8.5%	19.7%	45.1%	14.1%	5.6%	7.0%	0.59
S3	2.8%	0.0%	2.8%	94.4%	0.0%	0.0%	0.85
S4	1.4%	9.9%	4.2%	12.7%	71.8%	0.0%	0.79
REM	0.0%	1.4%	1.4%	0.0%	1.4%	95.8%	0.94
**Cohen’s kappa (±kappa error) = 0.7887 ± 0.0221**							
**Overall Accuracy = 82.4%**							

**Table 15:** Confusion matrix corresponding to sleep stage classification of narcolepsy patients with unbalanced data using the EBT classifier with 10-fold CV.

CT-4: Narcolepsy (Unbalanced Data)							
True Class	Predicted Class						F1 Score
	Wake	S1	S2	S3	S4	R	
Wake	91.0%	1.7%	4.3%	0.1%	0.2%	2.8%	0.87
S1	29.6%	21.3%	17.9%	0.0%	0.3%	30.9%	0.30
S2	3.9%	1.2%	84.0%	3.4%	0.6%	7.0%	0.80
S3	1.5%	0.0%	35.1%	50.6%	11.6%	1.3%	0.58
S4	0.7%	0.0%	5.1%	9.2%	85.0%	0.0%	0.86
REM	5.5%	2.0%	10.1%	0.1%	0.0%	82.4%	0.81
**Cohen’s kappa (±kappa error) = 0.7301 ± 0.0070**							
**Overall Accuracy = 79.3%**							

**Table 16:** Confusion matrix corresponding to sleep stage classification of narcolepsy patients with balanced data using the EBT classifier with 10-fold CV.

CT-4: Narcolepsy (Balanced Data)							
True Class	Predicted Class						F1 Score
	Wake	S1	S2	S3	S4	R	
Wake	87.0%	5.1%	3.0%	0.1%	0.2%	4.6%	0.89
S1	0.3%	99.7%	0.0%	0.0%	0.0%	0.0%	0.91
S2	3.5%	5.7%	71.7%	10.9%	1.0%	7.3%	0.78
S3	0.2%	0.0%	2.0%	95.8%	1.9%	0.0%	0.91
S4	0.1%	0.1%	0.4%	3.1%	96.3%	0.0%	0.97
REM	5.0%	8.6%	7.0%	0.3%	0.1%	79.0%	0.83
**Cohen’s kappa (±kappa error) = 0.8588 ± 0.0052**							
**Overall Accuracy = 88.2%**							

**Table 17:** Confusion matrix corresponding to sleep stage classification of nocturnal frontal lobe epilepsy patients with unbalanced data using the EBT classifier with 10-fold CV.

CT-5: NFLE (Unbalanced Data)							
True Class	Predicted Class						F1 Score
	Wake	S1	S2	S3	S4	R	
Wake	86.0%	2.5%	8.0%	0.1%	0.3%	3.1%	0.81
S1	42.3%	23.5%	18.6%	0.3%	0.0%	15.3%	0.34
S2	2.2%	0.4%	87.7%	3.9%	1.2%	4.7%	0.81
S3	0.2%	0.0%	50.3%	34.1%	15.1%	0.2%	0.43
S4	0.1%	0.0%	5.3%	8.1%	86.5%	0.0%	0.86
REM	3.1%	0.7%	16.3%	0.5%	0.3%	79.1%	0.81
**Cohen’s kappa (±kappa error) = 0.6914 ± 0.0035**							
**Overall Accuracy = 77.5%**							

**Table 18:** Confusion matrix corresponding to sleep stage classification of Nocturnal frontal lobe epilepsy patients with balanced data using the EBT classifier with 10-fold CV.

CT-5: NFLE (Balanced Data)							
True Class	Predicted Class						F1 Score
	Wake	S1	S2	S3	S4	R	
Wake	93.5%	3.3%	1.4%	0.1%	0.1%	1.6%	0.93
S1	0.0%	100.0%	0.0%	0.0%	0.0%	0.0%	0.96
S2	3.2%	2.2%	68.8%	15.1%	1.9%	8.9%	0.73
S3	0.2%	0.1%	9.1%	84.2%	6.1%	0.3%	0.80
S4	0.1%	0.0%	1.6%	9.%	89.1%	0.0%	0.90
REM	3.2%	2.2%	8.6%	1.3%	0.4%	84.3%	0.86
**Cohen’s kappa (±kappa error) = 0.8395 ± 0.0025**							
**Overall Accuracy = 86.6%**							

**Table 19:** Confusion matrix corresponding to sleep stage classification of periodic leg movement patients with unbalanced data using the EBT classifier with 10-fold CV.

CT-6: PLM (Unbalanced Data)							
True Class	Predicted Class						F1 Score
	Wake	S1	S2	S3	S4	R	
Wake	83.4%	4.6%	9.7%	0.3%	0.3%	1.7%	0.84
S1	32.3%	22.6%	25.2%	0.4%	0.0%	19.5%	0.28
S2	3.5%	1.3%	83.8%	7.2%	0.4%	3.7%	0.82
S3	0.5%	0.0%	26.0%	59.8%	13.2%	0.5%	0.61
S4	0.2%	0.0%	1.4%	15.1%	83.2%	0.2%	0.84
REM	1.4%	0.9%	8.8%	0.7%	0.2%	88.1%	0.87
**Cohen’s kappa (±kappa error) = 0.7296 ± 0.0061**							
**Overall Accuracy = 78.0%**							

**Table 20:** Confusion matrix corresponding to sleep stage classification of periodic leg movement patients with balanced data using the EBT classifier with 10-fold CV.

CT-6: PLM (Balanced Data)							
True Class	Predicted Class						F1 Score
	Wake	S1	S2	S3	S4	R	
Wake	89.5%	4.3%	4.3%	0.3%	0.1%	1.5%	0.90
S1	0.0%	100.0%	0.0%	0.0%	0.0%	0.0%	0.96
S2	5.9%	2.9%	69.5%	15.1%	1.0%	5.6%	0.72
S3	0.6%	0.1%	11.4%	80.0%	7.6%	0.3%	0.78
S4	0.0%	0.0%	1.0%	8.7%	90.3%	0.0%	0.91
REM	3.2%	1.7%	7.6%	1.3%	0.3%	85.8%	0.89
**Cohen’s kappa (±kappa error) = 0.8301 ± 0.0048**							
**Overall Accuracy = 85.8%**							

**Table 21:** Confusion matrix corresponding to sleep stage classification of REM behavioural disorder patients with unbalanced data using the EBT classifier with 10-fold CV.

CT-7: RBD (Unbalanced Data)							
True Class	Predicted Class						F1 Score
	Wake	S1	S2	S3	S4	R	
Wake	87.9%	1.1%	8.3%	0.2%	0.2%	2.2%	0.84
S1	29.0%	36.2%	24.2%	0.4%	0.0%	10.2%	0.47
S2	6.0%	1.0%	79.6%	5.5%	1.4%	6.5%	0.73
S3	1.4%	0.0%	31.6%	51.6%	12.3%	3.2%	0.56
S4	0.3%	0.0%	4.1%	14.3%	80.5%	0.9%	0.80
REM	9.1%	1.0%	31.6%	3.3%	0.8%	54.1%	0.61
**Cohen’s kappa (± kappa error) = 0.6372 ± 0.0039**							
**Overall Accuracy = 71.9%**							

**Table 22:** Confusion Matrix corresponding to sleep stage classification of REM behavioural disorder patients with balanced data using the EBT classifier with 10-fold CV.

CT-7: RBD (Balanced Data)							
True Class	Predicted Class						F1 Score
	Wake	S1	S2	S3	S4	R	
Wake	84.1%	4.9%	5.5%	0.7%	0.2%	4.7%	0.84
S1	0.1%	99.8%	0.1%	0.0%	0.0%	0.1%	0.94
S2	6.9%	4.7%	59.7%	13.0%	2.3%	13.5%	0.64
S3	0.8%	0.1%	8.8%	79.9%	7.9%	2.5%	0.78
S4	0.1%	0.0%	0.4%	6.3%	92.7%	0.6%	0.91
REM	7.3%	3.3%	13.2%	5.5%	1.1%	69.6%	0.73
**Cohen’s kappa (±kappa error) = 0.7715 ± 0.0031**							
**Overall Accuracy = 81.0%**							

**Table 23:** Confusion matrix corresponding to sleep stage classification of sleep-breathing disorder patients with unbalanced data using the EBT classifier with 10-fold CV.

CT-8: SBD (Unbalanced Data)							
True Class	Predicted Class						F1 Score
	Wake	S1	S2	S3	S4	R	
Wake	81.4%	9.1%	8.5%	0.2%	0.0%	0.8%	0.76
S1	34.9%	29.4%	34.2%	0.0%	0.4%	1.1%	0.35
S2	3.8%	3.4%	89.0%	2.0%	1.1%	0.8%	0.83
S3	0.0%	0.0%	60.3%	22.8%	17.0%	0.0%	0.31
S4	0.3%	0.0%	7.1%	8.0%	84.7%	0.0%	0.84
REM	6.5%	5.1%	25.6%	0.9%	3.7%	58.1%	0.70
**Cohen’s kappa (± kappa error) = 0.6276 ± 0.0117**							
**Overall Accuracy = 74.3%**							

**Table 24:** Confusion matrix corresponding to sleep stage classification of sleep-breathing disorder patients with balanced data using the EBT classifier with 10-fold CV.

CT-8: SBD (Balanced Data)							
True Class	Predicted Class						F1 Score
	Wake	S1	S2	S3	S4	R	
Wake	78.1%	14.2%	4.6%	1.0%	0.2%	1.9%	0.83
S1	5.2%	91.0%	2.3%	0.0%	0.8%	0.0%	0.85
S2	4.8%	8.8%	67.3%	12.7%	2.3%	4.2%	0.75
S3	0.0%	0.0%	2.9%	95.4%	1.7%	0.0%	0.88
S4	0.4%	0.2%	0.8%	5.8%	92.5%	0.2%	0.94
REM	0.4%	0.4%	1.3%	0.4%	0.4%	97.1%	0.95
**Cohen’s kappa (±kappa error) = 0.8429 ± 0.0075**							
**Overall Accuracy = 86.9%**							

**Table 25:** Confusion matrix corresponding to sleep stage classification of all disorders combined with unbalanced data using the EBT classifier with 10-fold CV.

CT-9: All Disordered (Unbalanced Data)							
True Class	Predicted Class						F1 Score
	Wake	S1	S2	S3	S4	R	
Wake	88.9%	1.3%	7.5%	0.1%	0.1%	2.1%	0.84
S1	39.7%	21.4%	24.2%	0.1%	0.1%	14.5%	0.31
S2	4.6%	0.6%	85.4%	3.6%	1.0%	4.9%	0.78
S3	1.1%	0.0%	42.5%	42.1%	12.9%	1.3%	0.50
S4	0.1%	0.0%	6.0%	10.2%	83.4%	0.2%	0.84
REM	6.0%	0.9%	22.3%	1.4%	0.3%	69.1%	0.74
**Cohen’s kappa (± kappa error) = 0.6780 ± 0.0021**							
**Overall Accuracy = 75.6%**							

**Table 26:** Confusion matrix corresponding to sleep stage classification of all disordered patients with balanced data using the EBT classifier with 10-fold CV.

CT-9: All disordered (Balanced Data)							
True Class	Predicted Class						F1 Score
	Wake	S1	S2	S3	S4	R	
Wake	87.7%	4.9%	3.8%	0.6%	0.2%	2.9%	0.87
S1	0.1%	99.8%	0.1%	0.0%	0.0%	0.1%	0.94
S2	6.7%	3.3%	65.6%	12.8%	1.4%	10.2%	0.71
S3	0.6%	0.0%	8.0%	85.4%	5.1%	0.9%	0.82
S4	0.1%	0.1%	0.9%	6.9%	91.9%	0.1%	0.92
REM	5.7%	3.5%	10.0%	2.9%	0.4%	77.5%	0.81
**Cohen’s kappa (± kappa error) = 0.8482 ± 0.1667**							
**Overall Accuracy = 84.8%**							

**Table 27:** Confusion matrix corresponding to sleep stage classification of all subjects combined (healthy + seven disordered) with unbalanced data using the EBT classifier with 10-fold CV.

CT-10: All Subjects Combined (Unbalanced Data)							
True Class	Predicted Class						F1 Score
	Wake	S1	S2	S3	S4	R	
Wake	87.8%	1.5%	7.9%	0.2%	0.1%	2.4%	0.83
S1	39.2%	21.4%	24.2%	0.1%	0.1%	15.0%	0.31
S2	4.7%	0.6%	84.4%	3.7%	1.1%	5.5%	0.78
S3	1.2%	0.0%	43.4%	41.1%	13.1%	1.2%	0.49
S4	0.2%	0.0%	5.9%	9.6%	84.0%	0.2%	0.84
REM	5.9%	0.8%	22.5%	1.3%	0.4%	69.1%	0.73
**Cohen’s kappa (± kappa error) = 0.6697 ± 0.0020**							
**Overall Accuracy = 75.5%**							

**Table 28:** Confusion matrix corresponding to sleep stage classification of all patients combined with balanced data using the EBT classifier with 10-fold CV.

CT-10: All Subjects Combined (Balanced Data)							
True Class	Predicted Class						F1 Score
	Wake	S1	S2	S3	S4	R	
Wake	88.1%	5.0%	3.5%	0.4%	0.1%	2.9%	0.88
S1	0.0%	100%	0.0%	0.0%	0.0%	0.0%	0.94
S2	6.2%	3.0%	66.5%	12.5%	1.4%	10.4%	0.70
S3	0.6%	0.1%	6.9%	87.5%	4.4%	0.6%	0.84
S4	0.1%	0.0%	0.8%	6.6%	92.3%	0.1%	0.93
REM	5.5%	3.2%	10%	2.8%	0.5%	78%	0.81
**Cohen’s kappa (± kappa error) = 0.8214 ± 0.0015**							
**Overall Accuracy = 85.1%**							

**Table 29:** Sleep stage-wise epochs and accuracy (before balancing) using the EBT classifier with 10-fold CV.

Sleep Stage	All Subjects Combined		Healthy Group		Sleep-Disordered Group	
	Epochs	Epochs (in %)	Epoch	Epochs (in %)	Epoch	Epochs (in %)
**Wake**	15,841	19.64	445	7.34	15,396	20.65
**S1**	3519	4.36	280	4.62	3239	4.34
**S2**	28,628	35.50	2172	35.82	26,456	35.48
**S3**	8804	10.92	573	9.45	8231	11.04
**S4**	10,188	12.63	1184	19.53	9004	12.07
**REM**	13,687	16.97	1409	23.24	12,278	16.45
**Total**	80,667	100.00	6063	100.00	74,604	100.00
**Accuracy**	**75%**		**78.8%**		**75.6%**	

**Table 30:** Sleep stage-wise epochs and accuracy (after balancing) using the EBT classifier with 10-fold CV.

Sleep Stage	All Subjects Combined		Healthy Group		Disordered-Group	
	Epochs	Epochs (in %)	Epoch	Epochs (in %)	Epoch	Epochs (in %)
**Wake**	14,000	16.66	1000	16.66	12,500	16.66
**S1**	14,000	16.66	1000	16.66	12,500	16.66
**S2**	14,000	16.66	1000	16.66	12,500	16.66
**S3**	14,000	16.66	1000	16.66	12,500	16.66
**S4**	14,000	16.66	1000	16.66	12,500	16.66
**REM**	14,000	16.66	1000	16.66	12,500	16.66
**Total**	84,000	100	6000	100	75,000	100
**Accuracy**	**85.1%**		**87.9%**		**84.8%**	

## Data Availability

Data used in this work is open-source and publicly available on PhysioNet.
